# Supplementary material for: MetaRibo-Seq measures translation in microbiomes
Source: Nat Commun. 2020 Jun 29;11:3268. doi: 10.1038/s41467-020-17081-z (PMC7324362; doi:10.1038/s41467-020-17081-z)
Supplement: Supplementary file 10 — Supplementary Data 7 [file 41467_2020_17081_MOESM10_ESM.zip › File2/Confidence_High_Taxonomy/108687_out.krona.html]

Javascript must be enabled to view this page.

members
magnitude
magnitudeUnassigned
count
unassigned
taxon
rank

108687\_out

41

41
2
superkingdom

phylum
1239
41

class
186801
41

186802
41
order

41
541000
family

genus
1263
41


SRS012273\_contig\_number\_contig-100\_347.182162SRS013098\_contig\_number\_42262SRS013951\_contig\_number\_contig-100\_18547.116809SRS014235\_contig\_number\_46328SRS015578\_contig\_number\_1497SRS015579\_contig\_number\_contig-100\_10163.58239SRS015663\_contig\_number\_22406SRS015782\_contig\_number\_contig-100\_17231.61210SRS015854\_contig\_number\_5167SRS015890\_contig\_number\_24894SRS018984\_contig\_number\_28967SRS019601\_contig\_number\_contig-100\_998.124291SRS019685\_contig\_number\_contig-100\_1825.186866SRS020233\_contig\_number\_49146SRS022071\_contig\_number\_440SRS024492\_contig\_number\_14958SRS048060\_contig\_number\_3979SRS048164\_contig\_number\_contig-100\_534.114650SRS050752\_contig\_number\_28599SRS051031\_contig\_number\_29695SRS051610\_contig\_number\_8890SRS052697\_contig\_number\_38676SRS053335\_contig\_number\_1503SRS062701\_contig\_number\_5974SRS063985\_contig\_number\_contig-100\_30249.70069SRS075821\_contig\_number\_1612SRS075984\_contig\_number\_1414SRS097920\_contig\_number\_contig-100\_11440.49803SRS097958\_contig\_number\_contig-100\_20117.20118SRS098571\_contig\_number\_41356SRS142542\_contig\_number\_23523SRS143148\_contig\_number\_24134SRS143181\_contig\_number\_13512SRS143722\_contig\_number\_contig-100\_6536.6537SRS143991\_contig\_number\_contig-100\_21492.72816SRS146764\_contig\_number\_22830SRS147039\_contig\_number\_contig-100\_12207.12208SRS147766\_contig\_number\_7564SRS148159\_contig\_number\_40664SRS148784\_contig\_number\_contig-100\_6011.44379SRS971275\_contig\_number\_25835
46228
41
species
